# Evidence of Cross-Cultural Consistency of the S-Five Model for Misophonia: Psychometric Conclusions Emerging From the Mandarin Version

**DOI:** 10.3389/fpsyg.2022.879881

**Published:** 2022-07-26

**Authors:** Silia Vitoratou, Jingxin Wang, Chloe Hayes, Qiaochu Wang, Pentagiotissa Stefanatou, Jane Gregory

**Affiliations:** ^1^Psychometrics and Measurement Lab, Department of Biostatistics and Health Informatics, Institute of Psychiatry, Psychology and Neuroscience, King’s College London, London, United Kingdom; ^2^First Department of Psychiatry, Eginition Hospital, Medical School, National and Kapodistrian University of Athens, Athens, Greece; ^3^Department of Experimental Psychology, University of Oxford, Oxford, United Kingdom; ^4^Oxford Health Specialist Psychological Interventions Centre, Oxford Health NHS Foundation Trust, Oxford, United Kingdom

**Keywords:** misophonia, S-Five, China, Mandarin, psychometrics

## Abstract

Misophonia is a disorder generally characterised by a decreased tolerance to everyday sounds. Although research is increasing in misophonia, a cross-cultural validation of a psychometric tool for measuring misophonia has not been evaluated. This study investigated the validity of the S-Five multidimensional model of the misophonic experience in a sample of Chinese participants. The S-Five was translated in a forward-backward method to Mandarin to establish a satisfactory translation. The translation was also independently back translated to English, with no significant differences when compared to the original S-Five. Through exploratory factor analysis, using responses from 256 Chinese individuals, the five dimensions (internalising appraisals, externalising appraisals, perceived threat and avoidance behaviour, outbursts, and impact on functioning) were replicated, indicating the cross-cultural uniformity of the experience of misophonia as captured by the S-Five. That is, current results point to the stability of the manifestation of misophonia across cultures, seen here for the first time in the literature. By design, the S-Five items were developed to reflect sound sensitivities in a manner that is not specific or matching to individuals of a certain age, gender, ethnicity, nationality, socio-economic status, and educational level. Testimonial to this fact is not only the replication of the five factors, but also the replication of the evidence towards satisfactory psychometric properties (reliability and validity) of the scale. Based on the results of this study, the S-Five is a psychometrically robust tool to be used within the Chinese population.

## Introduction

Misophonia is characterised by decreased tolerance to everyday sounds (Jastreboff and Jastreboff, 2001) and, by consensus, is recognised as a disorder (Swedo et al., 2021). Trigger sounds have been identified to broadly cluster into the three groups of eating sounds, nose/throat sounds and environmental sounds (Vitoratou et al., 2021a), with decreased sound tolerance to eating sounds appearing to be at the centre of the disorder (Jager et al., 2020; Swedo et al., 2021; Vitoratou et al., 2021a). Reactions and responses to sounds experienced in misophonia are varied and include emotional, physiological, and behavioural responses. It has been commonly reported that primary feelings such as anger and disgust are experienced (Edelstein et al., 2013; Schröder et al., 2013; Kumar et al., 2017; Jager et al., 2020), alongside unpleasant physiological changes, including an increased heart rate, muscle tension, pain and sweating (Edelstein et al., 2013; Johnson et al., 2013). Misophonia can have a significant impact on a person’s social and occupational functioning (Schröder et al., 2013; Rouw and Erfanian, 2018). Avoidance behaviours, social withdrawal (Johnson et al., 2013; Schneider and Arch, 2015; Hocaoglu, 2018; Muller et al., 2018; Singer, 2018; Alekri and Al Saif, 2019) and, for some, aggression (Reid et al., 2016; Hocaoglu, 2018; Alekri and Al Saif, 2019; Jager et al., 2020) are also frequently reported.

There is currently limited literature available on misophonia outside of western cultures. Two studies have evaluated the symptoms and clinical correlates of misophonia within Asian cultures. One study investigated the disorder within Chinese undergraduate students (Zhou et al., 2017) and another within Singaporean psychiatric patients (Quek et al., 2018). Zhou et al. (2017) found that 6% of respondents reported clinically significant levels of misophonia, with 17% endorsing a sensitivity (selecting “often” or “always” on the rating scale) to eating sounds, 18% to nasal sounds and 13% to environmental sounds. This study used the Misophonia Questionnaire (MQ; Wu et al., 2014), which has not undergone a full psychometric analysis. The MQ contains two factors: sensitivity to sounds compared to other people, as well as emotional and behavioural responses to those sounds. It does not capture some of the other aspects of misophonia reported in the literature, such as loss of control (Jager et al., 2020) and appraisals of oneself (Rouw and Erfanian, 2018) and of others (Edelstein et al., 2013).

Another questionnaire, MisoQuest (Siepsiak et al., 2020), was developed to assess the presence or absence of misophonia, based on the misophonia diagnostic criteria proposed by Schröder et al. (2013). It contains 14 items and measures misophonia from reactions to specific sounds, occurrence of emotions, controlling emotional reactions, attitudes toward reactions, avoidance, and daily dysfunction. MisoQuest has shown satisfactory psychometric properties but is not designed to capture severity of misophonic traits. The Duke Misophonia Questionnaire (DMQ; Rosenthal et al., 2021) was developed as a tool for assessing the complexities in symptom severity, impairment to functioning and coping mechanisms in misophonia. Composite scores can be calculated separately for symptoms and coping, rather than an overall score for misophonia severity, drawn from all subscale scores.

The S-Five tool, for measuring the latent trait of misophonia severity, was developed in large study (*n* = 828) initiated in English-speaking individuals who identify with the condition (Vitoratou et al., 2021b). Four waves of sampling, more than 80 initial items and several thousand of responses, concluded with a 25-item scale which reflects five dimensions of the misophonic experience, with excellent psychometric properties. The five factors that emerged were: emotional threat (sense of feeling trapped or helpless if unable to get away from sounds), internalising appraisals (tendency to see oneself as a bad or angry person for reacting to sounds) externalising appraisals (tendency to blame the person for making the sound), outbursts (fear of having, or actually displaying, aggressive outburst) and impact (current and future limitations in life from misophonia). The factor structure was subsequently replicated in a large sample (*n* = 772), representative of the UK population (Vitoratou et al., 2022). Individuals who identified with having misophonia had higher mean scores for threat factor than other factors (Vitoratou et al., 2021b). Meanwhile, in the general population, externalising appraisals was the factor most highly endorsed (Vitoratou et al., 2022). Within both populations, the S-Five subscales had an alpha of a least 0.83 (Vitoratou et al., 2021b, 2022). In both studies, misophonia severity was associated with increased symptoms of depression and anxiety.

The S-Five has a supplementary trigger checklist, capturing the nature and intensity of the emotional response to sounds (Vitoratou et al., 2021b, 2022), in a flexible format which allows modifications of the trigger sounds list and the response types, to accommodate advances made in the literature of misophonia research. Loud eating was the sound rated with the highest intensity of negative reaction in both the UK general population (Vitoratou et al., 2022) and by individuals identifying with having the condition (Vitoratou et al., 2021b).

The current study aimed to evaluate the five-factor model of the experience of misophonia in a non-clinical Chinese sample using the S-Five translated into Mandarin. We aimed to test the cross-cultural robustness of the five dimensions of the S-Five, evaluate the measurement invariance with regards to age and gender, examine the reliability (consistency and stability) and concurrent validity. We hypothesised that symptoms of depression and anxiety would be positively associated with symptoms of misophonia. With respect to trigger sounds, we hypothesised that loud chewing would be rated as causing the most intense negative reaction.

## Materials and Methods

### Recruitment

Inclusion criteria followed being aged 18 years and over and fluent in Mandarin. Exclusion criteria were the presence of a severe learning or intellectual disability, as per self-disclosure of such a disability. A participants’ information sheet was available at the beginning of the survey and consent was granted before completing the questionnaires online (ethics approval reference RESCM-19/20–11,826).

Recruitment was done using a snowball sampling technique *via* social media in China (Wechat Moment, Weibo & Douban), as well as *via* Twitter, Reddit, and the Fortnightly Recruitment Circular at King’s College London. Data collection took part between January and September 2021, including the retest study. Participants who finished the S-Five 25-item measurement scale were offered a chance to win an e-voucher at the end of the survey.

Retest data were collected between two and four weeks of an individual’s first assessment. The opportunity to take part in the retest study was presented at the end of the survey, to which participants were directed to a separate survey to enter their email addresses, to maintain anonymity. A total of 48 participants received the test–retest survey link *via* email and those that completed the survey were offered the chance to win an e-voucher again.

The e-voucher, in both surveys, was for an online video membership worth ¥130 (~£15), and SPSS random selection was used for establishing those who won. Those who partially completed the surveys were not offered the chance to win the e-voucher.

### Measures

The online survey included demographic questions, such as age, gender, ethnicity, education level, occupation, country of birth, and countries of residence in both past and present. The survey also asked whether the individual had any formal diagnoses on mental health conditions (including mood, anxiety, psychotic, personality, trauma, eating and substance abuse disorders), audiological conditions (e.g., tinnitus) and neurodevelopmental conditions (e.g., autism). Participants were asked whether they were aware of the term misophonia and whether they identified as having misophonia. Attention check questions have been used throughout the survey to ensure the quality of responses (e.g., Please slide the bar to option ‘2’ for us to ensure the validity of the responses). Responses which did not meet the requirement of the attention check questions or failed to respond to more than 3 of the 25 S-Five items were removed to ensure engagement with the study (*n* = 60). The following self-report questionnaires were also included.

#### Selective Sound Sensitivity Syndrome Scale

The Selective Sound Sensitivity Syndrome Scale (S-Five) is a 25-item measurement scale which assess the severity of misophonia (Vitoratou et al., 2021b). Each item is rated on an 11-point scale from 0 (not at all true) to 10 (completely true). The items are presented in the appendix in both English and Mandarin.

The S-Five trigger checklist (S-Five-T; see appendix for the English and Mandarin versions) was designed to capture the nature and intensity of a range of trigger sounds. The S-Five-T is flexible by design, in that it allows for adjustment of the number of triggers used. The current study used the 37 trigger sounds presented in the original validation study for the S-Five (Vitoratou et al., 2021b). The original options for emotional reactions were also used (no feeling, irritation, distress, disgust, anger, panic, other feeling: negative, and other feeling: positive). Respondents select their main emotional reaction to each trigger item and then rate the intensity (henceforth trigger intensity) of that reaction, from 0 (does not bother me at all) to 10 (unbearable/causes suffering). Four indices can be computed: (1) the trigger count (TC), which is the total number of triggers endorsed (i.e., where a negative reaction is selected) by a respondent (takes values from 0 to 37 in the current list), (2) the reaction count (RC), the number of times each particular reaction type is endorsed, counted across triggers in a single respondent (takes values from 0 to 37 in the current list), (3) the frequency/intensity of reactions score (FIRS) is the total value of the intensity items of all endorsed triggers (takes values from 0 to 370 in the current list), and (4) the relative intensity of reactions score (RIRS) which gives an estimate of the intensity of reactions to triggers, relative to the number of triggers reported (takes values from 0 to 100 in the current list). It is computed by dividing the FIRS index by the TC index. The S-Five and S-Five-T were translated by the research team for use in the present study.

#### Amsterdam Misophonia Scale

The Amsterdam Misophonia Scale (A-MISO-S) is a 6-item measure of misophonia adapted from a clinician-rated tool, the Yale-Brown Obsessive–Compulsive Scale (YBOCS; Goodman et al., 1989; Schröder et al., 2013). Although it was designed as a clinician-rated tool, for the purposes of this study we administered it as a self-report measurement tool. The questions ask about misophonia in relation to time occupied, impact on functioning, level of distress, resistance of sounds, perceived control, and avoidance behaviour. The A-MISO-S, translated by the research team, had an alpha of 0.79 and an omega of 0.81 in this study.

#### Misophonia Questionnaire

The Misophonia Questionnaire (MQ) is a three-part self-report measure for misophonia (Wu et al., 2014). The Misophonia Symptoms Scale (MSYS; *α* = 0.70 and *ω* = 0.90) asks respondents to compare their sensitivity to specific triggers with others’ responses and the Misophonia Emotions and Behaviours Scale (MEBS; *α* = 0.89 and *ω* = 0.90) measures an individual’s responses to trigger sounds. The two subscales are combined to create the MQ total score. The Misophonia Severity Scale is a single item question, adapted from the NIMH Global Obsessive–Compulsive Scale (NIMH GOCS; Murphy et al., 1982), asking individuals to rate the severity of their sound sensitivity on a scale from 1 (minimal) to 15 (very severe), with a score greater than or equal to 7 said to indicate clinically significant symptoms. The MQ was translated by the research team for use in this study.

#### Patient Health Questionnaire-9

The Patient Health Questionnaire-9 (PHQ-9) was used to measure symptoms of depression (Kroenke et al., 2001). Items are rated on a 4-point ordinal scale, with a total score range of 0 to 27. We used a Mandarin version that has been validated in Chinese populations (Yeung et al., 2008). The reliability coefficients of PHQ-9 were *α* = 0.89 and *ω* = 0.89.

#### General Anxiety Disorder-7

The General Anxiety Disorder-7 (GAD-7) measures severity of anxiety symptoms (Spitzer et al., 2006). Each item is rated on a 4-point ordinal scale, with a total score ranging from 0 to 21. We used a Mandarin version that has been validated within Chinese populations (He et al., 2010). In this study, the GAD-7 had an *α* of 0.91 and *ω* of 0.92.

### Translation

The S-Five, developed in English, was translated into Mandarin for use in the Chinese population and then back-translated into English. Two authors (JW and QW), fluent in English and Mandarin, separately translated the S-Five, and the two versions were compared and revised accordingly. The co-adjusted version was translated back to English by a native Mandarin speaker, fluent in English. The back-translated version of the S-Five was compared to the original English version of the S-Five and a second co-adjusted version was produced. This version was again translated to English by the native Mandarin speaker. There were no significant differences between the final version of the translated S-Five and the original S-Five. Using the same method, the A-MISO-S and the MQ were translated to Mandarin for use in this study (please contact first author for the translated versions).

### Statistical Analysis

The latent structure of the S-Five was evaluated using exploratory factor analysis (EFA). The suitability of the data for use in factor analysis was first assessed using the anti-image correlations and the corresponding Kaiser-Meyer-Olkin (KMO) test for sampling adequacy (Kaiser, 1960; Kaiser and Rice, 1974) and Bartlett (1951) test of sphericity.

The factor extraction method implemented was maximum likelihood with robust standard errors in Mplus (MLR; Muthen and Muthén, 1998) due to skewness in the data, and the factors were allowed to correlate using the Oblimin rotation. Two criteria, based upon eigenvalues, were followed for identifying the number of factors to retain. First, the Guttman-Kaiser criterion (Guttman, 1954; Kaiser, 1960) suggests retaining about as many factors as the number of eigenvalues above 1 (factor variances) in the sample covariance matrix. Second, the parallel analysis criterion (Horn, 1965) compares the number of sample eigenvalues to those produced by 1,000 set of randomly simulated data, with the same number of observations and number of factors. The number of factors to retain is identified by the number of sample eigenvalues larger than the simulated data eigenvalues. Parallel analysis was carried out in Mplus under MLR estimator (Muthen and Muthén, 1998), the parallel analysis average eigenvalues and 95th percentile parallel analysis eigenvalues. The eigenvalues computed using the sample correlation matrix and the parallel analysis simulated data are presented graphically using Cattell (1966) scree plot.

Absolute and relative goodness of fit indices were used to evaluate the fit of the EFA suggested models. The indices reported and the criteria followed were the relative chi-square (relative 𝜒^2^: values close to 2 suggest a close fit; Hoelter, 1983), the Root Mean Square Error of Approximation (RMSEA: values <0.05 are required for close fit; Hu and Bentler, 1999), the Tucker-Lewis Index (TLI: values >0.95 suggest close fit; Bentler and Bonett, 1980), the Comparative Fit Index (CFI: values >0.95 are required for a close fit; Hu and Bentler, 1999) and the Standardized Root Mean Residual (SRMR: values <0.08 are needed for a good fit; Hooper et al., 2008). Model selection criteria were also considered, namely Akaike’s Information Criteria (AIC; Akaike, 1974) and Bayesian Information Criteria (BIC; Schwarz, 1978) were reported, for which a lower value indicates a better model.

The multiple indicator multiple causes model (MIMIC; Joreskog and Goldberger, 1975; Muthén, 1979) was used to assess measurement invariance in relation to gender and age. An item was considered measurement non-invariant when the effect of the exogenous variable (age or gender) on the item directly (hereafter direct effect or de) was statistically significant. The MIMIC model was preferred in this study to allow for testing the measurement invariance of the S-Five items in relation to gender and age, each adjusted (controlled) for the other. Cohen’s d (Cohen, 1988) was used for the effect sizes (small, medium, and large effects correspond to d = 0.2, 0.5, and 0.8, respectively).

The internal consistency of S-Five factors was evaluated by Cronbach (1951) alpha and McDonald (1999) Omega, for which values of *α* and *ω* >0.7 suggest satisfactory internal consistency. The alpha if item deleted and the item-total correlations (ITC), for which values between 0.3 and 0.8 were considered acceptable (Nunnally and Bernstein, 1994).

The test re-test reliability was evaluated, at item and factor level, by the intraclass correlations coefficient (ICC: two-way mixed effects with absolute agreement; Shrout and Fleiss, 1979) and the Psi Non-Parametric Concordance Coefficient (Psi; Kuiper and Hoogenboezem, 2019). The Psi coefficient value represents the probability that a value randomly drawn from the data matrix will fall outside of the difference between the measurement scores at each time point (Rothery, 1979). For acceptable test–retest reliability, values above 0.75 for both coefficients were expected, according to Koo and Li (2016).

Convergent and concurrent validity were established through correlating the S-Five with the two other measurements scales for misophonia (MQ and A-MISO-S). Discriminant validity was established by correlating the S-Five with the GAD-7 and PHQ-9. Hypothesis testing was carried out, with respect to linear relationships between the S-Five and age, and gender differences in S-Five scores.

The statistical software of Stata 16 (StataCorp, 2019), Mplus 8 (Muthen and Muthén, 1998) and R (R Core Team, 2020) were used to carry out the analysis.

## Results

### Descriptive Indices

The sample (*n* = 256) consisted of 186 females (71%) and 66 males (25%), with a mean age of 25 years (*sd* = 6.5; *n* = 251) which did not differ across genders (*p* > 0.05). Missing data was low. For instance, missingness for age was 2% (*n* = 4) and 1% (*n* = 3) for gender. Where missingness was present in the variables used in the analysis. Listwise deletion was used, thus sample sizes vary.

The majority of the sample, 154 people (60%), had completed an undergraduate degree and 161 (63%) were students at the time of completing the study. Most (88%) of participants were Han, the rest were from minority ethnic groups, including Uygur, Yi, Manchu, Tujia, Zhuang, Bai and Mongolian. All participants were born in China and lived there at the time of completing the survey.

With respect to reported mental health and audiological conditions, the most often reported were depression (5%), social anxiety (4%) and tinnitus (4%). In terms of misophonia, 85 participants (33%) stated they were aware of the term misophonia and 41 (16%) identified as having misophonia. Autonomous sensory meridian response (ASMR) was experienced by 42% of the sample and synaesthesia by 25% (28% were unsure).

The retest sample (*n* = 34) included 4 males (11.8%) and 30 females (88.2%), with an age range of 19 to 36 years old (*mean* = 23.5, *sd* = 3.44).

### S-Five Statements

#### Statement Responses

The descriptive indices of the 25 S-Five statements are presented in Table 1. The items more widely endorsed (higher mean/median) were those related to the *externalising* and *threat* factors. None of the items correlated significantly with age but there were score differences with respect to gender (Table 1). Interestingly, none of the items referring to the *externalising* and *threat* items factors differed across genders, while males scored significantly higher than females in almost all other items.

**Table 1:** Descriptive indices, associations with age and gender, factor analysis loadings to factors, and reliability indices of the 25 S-Five items (*N* = 225).

S-Five-E statements per factor	Mean (sd)	Median (Q1–Q3)	Mode (min–max)	Age rho (*p*)	Gender difference mean (se)^‡^	Loadings EFA	Psi (95% CI)	ICC
**Externalising**								
I06 Others avoid noises	7.0 (2.9)	8 (6–10)	10 (0–10)	−0.06 (0.369)	0.35 (0.40)	0.71	0.77 (0.70,1)	0.85
I13 Others not make sounds	5.7 (3.1)	6 (3–8)	6 (0–10)	−0.04 (0.547)	0.54 (0.44)	0.69	0.80 (0.72,1)	0.86
I16 Others selfish	5.5 (3.0)	6 (3–8)	7 (0–10)	0.06 (0.373)	0.81 (0.44)	0.81	0.83 (0.76,1)	0.87
I21 Others bad manners	5.4 (3.0)	6 (3–8)	6 (0–10)	−0.01 (0.912)	0.77 (0.42)	0.79	0.72 (0.65,1)	0.83
I25 Others disrespectful	6.0 (3.0)	7 (4–8)	7 (0–10)	−0.10 (0.132)	0.84 (0.42)	0.79	0.79 (0.71,1)	0.85
**Internalising**								
I05 Respect myself less	2.7 (2.9)	1 (0–5)	0 (0–10)	0.03 (0.659)	1.33^**^ (0.41)	0.78	0.81 (0.75,1)	0.86
I08 Unlikeable person	2.9 (3.0)	2 (0–5)	0 (0–10)	0.1 (0.113)	1.70^**^ (0.42)	0.78	0.84 (0.79,1)	0.87
I12 Angry person inside	3.5 (3.0)	3 (1–6)	0 (0–10)	0.02 (0.719)	0.63 (0.43)	+0.58	0.85 (0.79,1)	0.88
I18 Bad person inside	2.7 (2.8)	2 (0–5)	0 (0–10)	0.05 (0.465)	1.31^**^ (0.40)	0.80	0.78 (0.70,1)	0.85
I19 Dislike self	2.9 (3.0)	2 (0–5)	0 (0–10)	0.00 (0.979)	0.92^*^ (0.43)	0.85	0.81 (0.74,1)	0.86
**Impact**								
I01 Do not meet friends	2.1 (2.6)	1 (0–3)	0 (0–10)	0.06 (0.360)	0.81^*^ (0.37)	0.78	0.81 (0.75,1)	0.86
I09 Eventually isolated	2.7 (2.9)	1 (0–5)	0 (0–10)	0.08 (0.217)	0.96^**^ (0.41)	0.63	0.79 (0.72,1)	0.85
I14 Avoid places	2.7 (2.8)	2 (0–5)	0 (0–10)	0.03 (0.679)	0.78^*^ (0.40)	0.75	0.81 (0.75,1)	0.86
I15 Cannot do things	2.8 (2.8)	2 (0–5)	0 (0–10)	0.05 (0.433)	0.84^*^ (0.40)	0.81	0.77 (0.70,1)	0.85
I20 Limited job opportunities	2.6 (2.7)	2 (0–4)	0 (0–10)	0.02 (0.718)	0.63^*^ (0.39)	0.80	0.81 (0.73,1)	0.86
**Outburst**								
I04 Verbally aggressive	4.6 (3.0)	5 (2–7)	6 (0–10)	−0.01 (0.822)	0.94^*^ (0.43)	0.59	0.84 (0.78,1)	0.87
I17 Physically aggressive	2.7 (2.7)	2 (0–5)	0 (0–10)	0.01 (0.852)	1.04^**^ (0.39)	0.62	0.80 (0.73,1)	0.86
I22 Violence	2.9 (2.8)	2 (0–5)	0 (0–10)	0.00 (0.999)	1.06^**^ (0.40)	0.61	0.79 (0.73,1)	0.85
I23 Shout at people	3.6 (2.9)	3 (1–6)	0 (0–10)	0.02 (0.716)	1.20^**^ (0.41)	0.71	0.88 (0.82,1)	0.89
I24 Afraid of outburst	3.1 (3.0)	2 (0–5)	0 (0–10)	0.04 (0.569)	0.95^*^ (0.43)	0.62	0.86 (0.80,1)	0.88
**Threat**								
I02 Panic or explode	4.5 (3.2)	4 (2–7)	0 (0–10)	−0.08 (0.186)	0.34 (0.47)	0.81	0.85 (0.79,1)	0.88
I03 Feel helpless	4.4 (3.2)	5 (2–7)	0 (0–10)	−0.03 (0.592)	0.38 (0.46)	0.77	0.83 (0.76,1)	0.87
I07 Feel anxious	5.0 (3.2)	5 (2–7)	6 (0–10)	−0.11 (0.080)	−0.13 (0.45)	0.89	0.81 (0.73,1)	0.86
I10 Experience distress	5.6 (3.2)	6 (3–8)	10 (0–10)	−0.06 (0.382)	0.29 (0.45)	0.74	0.79 (0.71,1)	0.85
I11 Feel trapped	4.5 (3.1)	5 (2–7)	0 (0–10)	−0.06 (0.329)	0.27 (0.45)	0.83	0.81 (0.73,1)	0.86

Q1–Q3, first and third quartile; ICC, intraclass correlation coefficient; Psi, coefficient and 95% confidence interval; rho, Spearman’s correlation coefficient; ‡mean difference (se) male vs female comparison, value of *p* via Mann Whitney test. ^+^The item had a salient cross-loading (0.31) on the Outburst factor.

* *p* < 0.05;

** *p* < 0.01.

#### Dimensionality and Measurement Invariance

First, we established that the sample correlation matrix suggested the existence of latent vectors. The anti-image correlations were above 0.88 for all statements, the KMO was 0.94, and Bartlett’s test was significant (*χ*^2^ = 13,773,1, *df* = 300, *p* < 0.001). We therefore proceeded with exploratory factor analysis.

**Figure 1:**
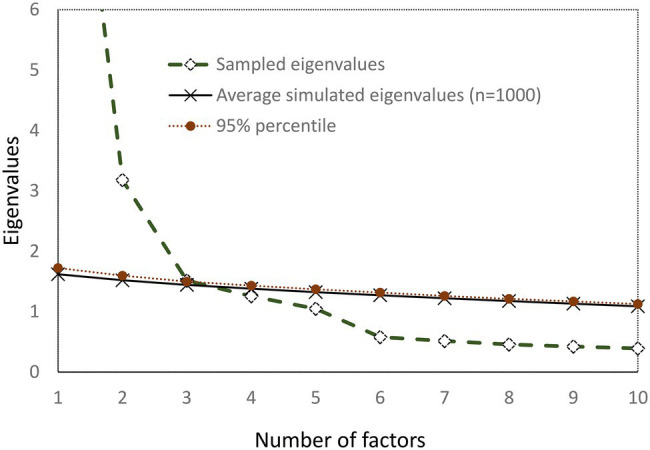
Scree plot of observed and simulated data (Parallel analysis).

The sample correlation matrix emerged five eigenvalues above 1 (12.1, 3.2, 1.5, 1.3, and 1.1) and hence the Kaiser-Guttman criterion points towards a five-factor structure, explaining 73% of the total variance. Parallel analysis, on the other hand, indicated that three factors should be extracted, as is depicted in the scree plot in Figure 1. The goodness-of-fit examination suggested that the three-factor model however did not fit the data adequately [rel *χ*^2^ = 4.3; RMSEA = 0.1 with 90% (0.107, 0.122), TLI = 0.81, CFI = 0.86, SRMR = 0.051, AIC: 27491.6, BIC: 27923.6]. The goodness-of-fit was improved for the four-factor model [rel *χ*^2^ = 3.02; RMSEA = 0.09 with 90% (0.081, 0.197), TLI = 0.89, CFI = 0.92, SRMR = 0.036, AIC: 27169.3, BIC: 27679.3], but close fit was only achieved in the 5 factor models [rel *χ*^2^ = 2.01; RMSEA = 0.063 with 90% (0.054, 0.072), TLI = 0.94, CFI = 0.97, SRMR = 0.020, AIC: 26960.8, BIC: 27545.1]. Increasing the factors to six led to a sixth factor with no loading larger than 0.3 (overfitting). Therefore, the five-factor solution was accepted in our data. The five factor solution loadings are presented in Table 2 (see appendix A3 for the full pattern matrix) and the assignment of the items to factors coincides completely with the original model found by Vitoratou et al. (2021b).

**Table 2:** Norms and reliability of the S-Five 5 factors and total scores (*N* = 255).

**Factor**	**Descriptive indices**					**Internal consistency**		**Stability**	
	**Mean (sd)**	**Median (Q1–Q3)**	**Mode (min–max)**	**Gender difference mean (sd)** ^**‡**^	**Age rho**	***α* /*ω***	**ITC**	**Psi (95% CI)**	**ICC**
Externalising	29.7 (12.4)	32 (22–38)	30 (0–50)	3.3 (1.747)	−0.03 (0.598)	0.88 /0.88	0.68–0.75	0.81 (0.74,1)	0.86
Internalising	14.7 (12.9)	11 (4–25)	0 (0–46)	5.88^**^ (1.814)	0.04 (0.546)	0.92 /0.92	0.72–0.84	0.85 (0.79,1)	0.88
Impact	12.9 (12.2)	7 (3–21)	0 (0–50)	4.01^*^ (1.745)	0.06 (0.316)	0.93 /0.93	0.81–0.84	0.81 (0.74,1)	0.86
Outburst	16.8 (12.3)	15 (6–27)	0 (0–50)	5.19^**^ (1.731)	0.00 (0.975)	0.93 /0.93	0.87–0.84	0.87 (0.81,1)	0.89
Threat	24.1 (14.1)	25 (12–35)	0 (0–50)	1.14 (2.016)	−0.09 (0.176)	0.90 /0.90	0.67–0.81	0.82 (0.75,1)	0.87
S-Five total	98.1 (50.9)	96 (56–135)	70 (0–232)	19.54^**^ (7.173)	−0.01 (0.926)	0.95 /0.95	0.36–0.77	0.88 (0.82,1)	0.89

sd, standard deviation; Q1–Q3, first and third quartile respectively; *α*, Cronbach’s alpha; *ω*, McDonald’s omega; ITC, item-total correlations; ICC, intraclass correlation coefficient (two-way mixed effects, absolute agreement).

‡ mean difference (standard error) male vs female comparison, value of *p* via Mann Whitney test. *p < 0.05 and **p < 0.01.

We proceeded with the evaluation of the measurement invariance of the tool with respect to gender and age using the MIMIC model. Adjusted for gender and the five latent dimensions, only one item was found to be non-invariant with respect to age, namely item I02 (‘If I cannot get away from certain noises, I am afraid I might panic or feel like I’ll explode’), being less endorsed on average as age increases (de = −0.04, *p* = 0.027). The direct effect was however very small (0.04 units on a scale of 0 to 10, for each additional year in age, that is 0.4 units between decades, Cohen’s *d* = −0.0043) and can be considered negligible. With respect to gender, men tend to endorse more often the same item (I02) compared to women of the same age and latent positions (de = −0.65, *p* = 0.015, Cohen’s *d* = −0.065). Finally, women tend to endorse more the item I08 (‘the way I react to certain noises makes me feel like I must be an unlikable person deep down’) compared to men of the same age and latent positions (de = 0.55, *p* = 0.019, Cohen’s *d* = −0.06). In all cases the effects were less than half a unit on an 11-unit rating scale, and as only two effects were identified in the case of gender and one in the case of age, it is reasonable to conclude that the S-Five scores are effectively measurement invariant with respect to those factors and therefore the assessment of structural invariance (factor score differences) is reasonably justified.

### S-Five Scores: Reliability and Validity

None of the S-Five factor scores were correlated with age in our sample (Table 2). While there were no gender differences in the scores of the externalising and threat factors, in all other factors men scored significantly higher than women.

With respect to internal consistency, alpha and omega were satisfactory within all factors (0.88 or higher; Table 2), while test–retest reliability was also satisfactory with ICC being larger than 0.86 for all S-Five scores.

Table 3 presents the correlations of the S-Five factor scores and total score with several measurement scales, namely, two misophonia scales (MQ and A-MISO-S), PHQ-9 and GAD-7. Evidence of convergent validity is demonstrated by moderately strong correlations between the S-Five total score and the MQ and A-MISO-R. With respect to the PHQ-9 and GAD-7, low to moderate positive correlations with the S-Five factors and total score were found. Intercorrelations between the S-Five factors ranged from 0.3 to 0.7 and, as expected moderate to strong correlations were identified (Table 3). Additional evidence of discriminative validity was demonstrated by a significantly higher score on all S-Five factors and S-Five total score for those self-identifying as having misophonia compared to those who did not self-identify as (for instance, S-Five total (*n* = 33) *mean* = 146.12, *sd* = 41.0 versus S-Five total (*n* = 159) *mean* = 88.65, *sd* = 49, t_53.8_ = 6.964, *p* < 0.001, respectively).

**Table 3:** Intercorrelations of the S-Five scores, and correlations with other measures (validity assessment).

	**Externalising**	**Internalising**	**Impact**	**Outburst**	**Threat**	**Total S-Five**
**S-Five (***N*** = 255)**						
Internalising	0.30					
Impact	0.27	0.71				
Outburst	0.40	0.70	0.68			
*Threat*	0.50	0.60	0.54	0.64		
Total	0.61	0.84	0.78	0.87	0.84	
**A-MISO-S (***N*** = 125)**						
Total	0.41	0.42	0.46	0.40	0.52	0.57
**MQ (***N*** = 118)**						
MSYS (*N* = 114)	0.41	0.42	0.33	0.31	0.43	0.47
MEBS (*N* = 105)	0.33	0.46	0.35	0.50	0.58	0.58
MSES (*N* = 118)	0.46	0.49	0.37	0.42	0.48	0.57
Total (*N* = 118)	0.48	0.43	0.33	0.41	0.50	0.54
**PHQ9 (***N*** = 130)**						
Total	0.31	0.35	0.22	0.24	0.30	0.35
**GAD7 (***N*** = 128)**						
Total	0.27	0.38	0.24	0.30	0.33	0.37

Correlations are Spearman’s rho and value of *p* < 0.01 in all cases. A-MISO-S, Amsterdam Misophonia Scale; MQ, Misophonia Questionnaire; MSYS, Misophonia Symptoms Scale; MEBS, Misophonia Emotions and Behaviours Scale; MSES, Misophonia Severity Scale; PHQ-9, Physical Health Questionnaire; GAD-7, Generalised Anxiety Disorder Assessment.

#### S-Five-T Scoring Instructions

The S-Five-T items and the scoring instructions are presented in the appendix (English and Mandarin). The norms of the S-Five-T are presented in Table 4.

**Table 4:** Norms and reliability of the S-Five-T scores (*N* = 78).

**S-Five RC (***N*** = 255)**	**Mean (sd)**	**Median (Q1-Q3)**	**Mode (min-max)**	**Gender difference mean (se)** ^**‡**^	**Age rho**
No feeling	19.7 (7.7)	18 (15–25)	16 (0–37)	0.59 (2.9)	−0.11 (0.335)
Irritation	4.9 (3.4)	4 (3–7)	3 (0–15)	−2.3^*^ (1.2)	0.25^*^ (0.030)
Distress	1.2 (1.8)	1 (0–2)	0 (0–11)	0.3 (0.7)	−0.165 (0.152)
Disgust	2.7 (2.7)	2 (1–4)	0 (0–11)	−0.7 (1.0)	0.24^*^ (0.037)
Anger	1.0 (1.7)	0 (0–1)	0 (0–10)	0.3^*^ (0.7)	0.08 (0.474)
Panic	1.9 (2.0)	1 (1–3)	1 (0–12)	0.8 (0.8)	−0.05 (0.663)
TC	15.0 (7.0)	15 (11–21)	12 (0–30)	−0.6 (2.6)	0.14 (0.231)
FIRS	79.2 (45.2)	75 (44–115)	75 (0–184)	−7.9 (11.1)	0. 11 (0.336)
RIRS	4.1 (1.6)	5 (4–6)	6 (1–8)	0.3 (0.6)	0.07 (0.549)

RC, response count; TC, total count; FIRS, frequency and intensity reaction count; RIRS, relative intensity of reactions score; sd, standard deviation; Q1 – Q3, first and third quartile; rho, Spearman’s correlation coefficient; ICC, intraclass correlation coefficient; Psi, coefficient and 95% confidence intervals.

‡ Mean difference (se) male vs female comparison, value of *p* via Mann Whitney test.

* *p* < 0.05 and ^**^p < 0.01.

#### Reaction Counts

On average, participants reported 20 out of 37 trigger sounds caused *no feeling* (Table 4). *Irritation* was the next highest reported reaction, with an average of 5 trigger sounds reported as causing this reaction. *Irritation* and *disgust* had small, significant positive correlations with age. In terms of gender, women scored significantly higher on the RC for *irritation*, while men scored higher on *anger*.

With respect to the RC scores, the intercorrelations varied between 0.2 and 0.7 (Table 5). All correlations were positive except for the *no feeling* count, for which all correlations with other variables were negative. Interestingly, disgust correlated only with *no feeling* and *irritation*. *Distress* had low correlations with all other RCs. The highest correlations emerged between *no feeling*, *anger* and *panic*. The total number of triggers reported was highly correlated with disgust and emerged similar coefficients with FIRS. RIRS on the contrary did not correlate with *disgust*, *anger* or *panic*.

**Table 5:** Intercorrelations of the S-Five, S-Five-T scores, and correlations with other measures (Spearman’s rho).

	**No feeling**	**Irritation**	**Distress**	**Disgust**	**Anger**	**Panic**	**TC**	**FIRS**	**RIRS**
**S-Five RC (***N*** = 81)**									
No feeling		−0.62^**^	−0.36^**^	−0.41^**^	−0.38^**^	−0.44^**^	−0.87^**^	−0.82	−0.44^**^
Irritation			0.25^*^	0.36^**^	0.19^*^	0.26^*^	0.63^**^	0.59^**^	0.33^**^
Distress				0.15	0.23^*^	0.26^*^	0.41^**^	0.39^**^	0.28^*^
Disgust					0.02	0.17	0.53^**^	0.45^**^	0.19
Anger						0.35^**^	0.38^**^	0.27^*^	0.11
Panic							0.40^*^	0.33^*^	0.12
TC								0.89^**^	0.37^**^
FIRS									0.70^**^
**S-Five Factors (***N*** = 78)**									
Externalising	−0.39^**^	0.39^**^	0.27^*^	0.14	0.26^*^	0.22	0.46^**^	0.37^**^	0.13
Internalising	−0.40^**^	0.30^**^	0.33^**^	0.06	0.38^**^	0.25^*^	0.46^**^	0.46^**^	0.33^**^
Impact	−0.40^**^	0.36^**^	0.26^*^	0.06	0.27^*^	0.12	0.41^**^	0.46^**^	0.40^**^
Outburst	−0.43^**^	0.35^**^	0.5^**^	0.09	0.41^**^	0.31^**^	0.51^**^	0.45^**^	0.27^*^
Threat	−0.48^**^	0.38^**^	0.48^**^	0.24^*^	0.45^**^	0.26^*^	0.55^**^	0.52^**^	0.32^**^
Total	−0.49^**^	0.44^**^	0.47^**^	0.16	0.47^**^	0.3^**^	0.58^**^	0.56^**^	0.36^**^
**A-MISO-S (***N*** = 73)**									
Total	−0.44^**^	0.35^**^	0.33^**^	0.16	^**^0.48	^*^0.27	^**^0.56	^**^0.51	^**^0.35
**MQ (***N*** = 68)**									
MSYS (*N* = 68)	−0.68^**^	0.45^**^	0.42^**^	0.10	0.36^**^	0.36^**^	0.68^**^	0.65^**^	0.43^**^
MEBS (*N* = 59)	−0.34^**^	0.21	0.38^**^	−0.02	0.46^**^	0.16	0.41^**^	0.46^**^	0.42^**^
MSES (*N* = 59)	−0.55^**^	0.32^*^	0.38^**^	0.05	0.54^**^	0.28^*^	0.58^**^	0.60^**^	0.43^**^
Total (*N* = 68)	−0.52^**^	0.42^**^	0.37^**^	0.08	0.49^**^	0.29^*^	0.59^**^	0.61^**^	0.47^**^
**PHQ9 (***N*** = 73)**									
Total	−0.41^**^	0.26^*^	0.24^*^	0.05	0.17	0.31^**^	0.40^**^	0.37^**^	0.22
**GAD7 (***N*** = 72)**									
Total	−0.49^**^	0.24^*^	0.27^*^	0.07	0.17	0.34^**^	0.46^**^	0.41^**^	0.24^*^

RC, response count; TC, total count; FIRS, frequency and intensity reaction count; RIRS, relative intensity of reactions score; rho, Spearman’s correlation coefficient; A-MISO-S, Amsterdam Misophonia Scale; MQ, Misophonia Questionnaire; MSYS, Misophonia Symptoms Scale; MEBS, Misophonia Emotions and Behaviours Scale; MSES, Misophonia Severity Scale; PHQ-9, Physical Health Questionnaire; GAD-7, Generalised Anxiety Disorder Assessment. *p < 0.05 and **p < 0.01.

The RC for *no feeling*, *irritation, distress* and *anger*, and total count had moderate correlations with the A-MISO-S and MQ total score. The PHQ-9 and the GAD-7 were significantly correlated with the RC *distress* and *panic* and TC, while both were negatively correlated with the reaction count of *no feeling*.

#### Intensity

Table 6 presents the norms for the 37 intensity items. The sounds which cause reactions with the higher intensity were lip smacking, baby crying, and repetitive sounds of barking or engine. The sounds with the least intensity in the reaction were certain words and accents, yawning, and normal eating. Three items had low positive correlations with age (repetitive barking, loud chewing and teeth sucking), while normal breathing had a low negative correlation with age. For one item, coughing, men scored higher than woman.

**Table 6:** Norms and reliability of the intensity items for the 37 S-Five-T sounds.

**Trigger sounds**	**Mean (sd)**	**Median (Q1-Q3)**	**Mode (min-max)**	**Average Gender difference** ^**‡**^	**Age rho**
Normal eating sounds	1.7 (3.0)	0 (0–2)	0 (0–10)	−0.91 (0.64)	−0.06 (0.468)
Certain letter sounds	0.5 (1.7)	0 (0–0)	0 (0–10)	−0.31 (0.53)	0.06 (0.537)
Mushy foods	1.2 (2.4)	0 (0–1)	0 (0–10)	−0.03 (0.78)	0.06 (0.542)
Sound of clipping nails	1.1 (2.1)	0 (0–1)	0 (0–8)	−0.44 (0.71)	0.02 (0.814)
Swallowing	0.8 (2.1)	0 (0–0)	0 (0–10)	−0.24 (0.72)	−0.02 (0.847)
Keyboard tapping	1.4 (2.2)	0 (0–3)	0 (0–8)	−0.34 (0.73)	0.1 (0.355)
Lip smacking	4.4 (3.6)	4 (0–7)	0 (0–10)	−1.59 (1.2)	0.06 (0.606)
Normal breathing	0.4 (1.5)	0 (0–0)	0 (0–8)	0.26 (0.5)	−0.29 (0.006)
Repetitive engine	3.8 (3.3)	4 (0–6)	0 (0–10)	−2.06 (1.09)	−0.08 (0.483)
Blocked nose	3.6 (2.9)	3 (0–6)	0 (0–9)	−0.49 (1.02)	0.04 (0.696)
Mobile phone	1.7 (2.6)	0 (0–3)	0 (0–10)	0.8 (0.92)	0.15 (0.175)
Repetitive coughing	3.5 (3.1)	3 (0–6)	0 (0–10)	^*^2.35 (1.06)	0.04 (0.702)
Humming	3.2 (2.8)	3 (0–6)	0 (0–10)	0.76 (0.99)	0.02 (0.865)
Repetitive sniffing	2.6 (3.0)	2 (0–5)	0 (0–10)	1.01 (1.12)	−0.06 (0.569)
Snoring	3.6 (3.4)	4 (0–7)	0 (0–10)	−0.05 (1.26)	0.11 (0.327)
Certain accents	1.5 (2.6)	0 (0–2)	0 (0–10)	−0.33 (0.97)	−0.05 (0.678)
Whistling sound	0.8 (2.0)	0 (0–0)	0 (0–10)	−0.17 (0.76)	0 (0.987)
Tapping	2.8 (3.0)	2 (0–6)	0 (0–10)	1.57 (1.09)	0.12 (0.263)
Rustling plastic or paper	1.5 (2.3)	0 (0–3)	0 (0–7)	−0.67 (0.88)	0.12 (0.263)
Chewing gum	3.1 (3.3)	2 (0–6)	0 (0–10)	−1.76 (1.22)	0.1 (0.349)
Footsteps	1.5 (2.6)	0 (0–3)	0 (0–10)	−0.7 (0.97)	−0.09 (0.409)
Hiccups	1.5 (2.5)	0 (0–3)	0 (0–10)	1.11 (0.94)	−0.08 (0.451)
Slurping	1.8 (2.9)	0 (0–3)	0 (0–10)	−1.22 (1.09)	0.11 (0.312)
Cutlery	2.3 (3.1)	0 (0–5)	0 (0–10)	−1.02 (1.17)	0.03 (0.797)
Sneezing	1.0 (2.1)	0 (0–0)	0 (0–7)	1.18 (0.78)	0.21 (0.065)
Certain words	0.5 (1.3)	0 (0–0)	0 (0–7)	0.46 (0.5)	−0.2 (0.071)
Kissing	1.1 (2.4)	0 (0–0)	0 (0–10)	−0.97 (0.91)	0.02 (0.858)
Joint cracking	0.9 (2.2)	0 (0–0)	0 (0–10)	−0.06 (0.81)	0.01 (0.921)
Muffled sounds	2.9 (3.0)	2 (0–5)	0 (0–10)	0.5 (1.14)	−0.04 (0.749)
Throat clearing	1.3 (2.6)	0 (0–2)	0 (0–10)	0.65 (0.96)	0.08 (0.497)
Baby crying	4.7 (3.4)	5 (1–7)	0 (0–10)	0.03 (1.25)	0.14 (0.221)
Repetitive barking	3.9 (3.1)	4 (1–6)	0 (0–10)	1.75 (1.14)	0.24 (0.036)
Loud chewing	3.3 (3.8)	2 (0–6)	0 (0–10)	−1.5 (1.41)	0.24 (0.038)
Clock ticking	2.1 (3.0)	0 (0–4)	0 (0–10)	−0.67 (1.12)	0.11 (0.322)
Crunching	0.7 (2.3)	0 (0–0)	0 (0–10)	−0.76 (0.85)	−0.27 (0.018)
Teeth sucking	2.8 (3.3)	1 (0–6)	0 (0–10)	−1.18 (1.22)	0.26 (0.021)
Yawning	0.4 (1.5)	0 (0–0)	0 (0–8)	−0.46 (0.57)	−0.18 (0.115)

sd, standard deviation; Q1 Q3, first and third quartile; rho, Spearman’s correlation coefficient; ICC, intraclass correlation coefficient; Psi, coefficient and 95% confidence intervals.

‡ Mean difference (se) male vs female comparison, value of *p* via Mann Whitney test. ^*^p < 0.05 and ^**^p < 0.01.

## Discussion

The purpose of this study was to evaluate the psychometric properties of the Mandarin version of the S-Five questionnaire. This was, to our knowledge, the first study to validate a self-reported multidimensional questionnaire for misophonia within this population. The psychometric analysis conducted concluded that the original five factor structure found in the general UK population (Vitoratou et al., 2022) and a large sample of English-speaking individuals who identify with the condition (Vitoratou et al., 2021b) was replicated for the Mandarin version. The scale was also found to be reliable (both in terms of internal consistency of each factor and stability in time), measurement invariant with respect to age and gender, and evidence of its validity emerged.

The original five dimensions (internalising appraisals, externalising appraisals, perceived emotional threat, outbursts, and impact on functioning) were fully and accurately reproduced in a sample derived from a population that not only speaks a different language but also belongs to an Asian culture. This highlights the consistency of the multidimensional experience of misophonia as captured by the S-Five. The S-Five items were designed to reflect sound sensitivities in a manner that is not specific or more matching to individuals of a certain age, gender, ethnicity, nationality socio-economic status and educational level. In this study, we see evidence that indeed the S-Five is robust cross culturally. Most importantly, the reproduction of the five factors in a Chinese sample in Mandarin points to the stability of the manifestation of misophonia across cultures, seen here for the first time in the literature.

The convergent validity of the S-Five was established through correlating the factors of the scale and total score with previously development measures of misophonia. The MQ and the A-MISO-S were significantly, positively and moderately correlated with the five factors of the S-Five and with the total score. Spearman’s rho coefficients were comparable to those found in previous S-Five validation studies (Vitoratou et al., 2021b, 2022). We note the moderate correlations with other scales measuring misophonia, which we propose is due to the broader construct of misophonia captured by the S-Five than the other measures used for construct validity, which are not multidimensional. Future studies could assess convergent validity with another multidimensional tool, for example the Duke Misophonia Questionnaire (Rosenthal et al., 2021), which had not been published at the time the present study was designed.

This study found that the sounds of baby crying and lip smacking had the highest average intensity of reaction. This is contrary to our hypothesis that the most intense reaction would be from the sound of loud eating, as was found in a UK general population study (Vitoratou et al., 2022) and in a sample of individuals identifying with having misophonia (Vitoratou et al., 2021b). Lip smacking was also reported as eliciting the most intense reaction in a Dutch study (Schröder et al., 2019). Further research is needed to clarify whether there are cross cultural differences in the types of sounds eliciting negative reactions in misophonia. It was interesting to note that in the present study, the reaction count of irritation was positively correlated with the other negative reactions and was low to moderately correlated with S-Five factors. This is not consistent with studies using UK samples, which have shown very low (Vitoratou et al., 2022) or even negative correlations (Vitoratou et al., 2021b) between irritation and other S-Five factors. Further research is needed to better understand these contrasting results.

The S-Five also importantly highlights that the reactions to such sounds may be influenced by gender. Female participants scored significantly higher on the RC irritation, while men scored higher on the RC anger. With regards to the S-Five, male respondents scored significantly higher on the internalising appraisals, impact on functioning and outburst factors, as well as the total score. This was in contrast to the finding that female respondents scored significantly higher on internalising in a UK sample of individuals identifying with having misophonia (Vitoratou et al., 2021a), and the finding that there was no significant gender difference on these factors in a UK representative sample (Vitoratou et al., 2022). Further research is needed using representative samples to determine whether there are any cross-cultural differences in the relationship between gender and misophonia symptoms.

We found a positive correlation between symptoms of misophonia and symptoms of depression and anxiety, which supports the findings of previous studies. Zhou et al. (2017) found that in a sample of Chinese college students, misophonic symptoms and severity of misophonic symptoms were correlated with anxiety. Similarly, Quek et al. (2018) found a positive association between the severity of anxiety and the severity of misophonic symptoms in Singaporean psychiatric patients.

There were several limitations that arose in this study. First, the sample collected cannot be considered a representative sample of the Chinese population. This limits the use of the findings in being unable to compute and evaluate populations norms for misophonia. Also, our data come from the general population, and therefore our findings might be different to those that would have emerged in a clinical sample. Future research should replicate this work in a sample of people with misophonia. A further limitation of the study was the self-reporting of co-occurring diagnoses and symptoms of anxiety and depression; future studies would benefit from structured clinical interviews to examine the relationship between disorders. Additionally, the S-Five has not yet been tested for discriminative validity in relation to other disorders of sound intolerance, such as tinnitus or hyperacusis, which needs to be addressed in future research. Because of these limitations, it is unknown to what extent the S-Five assesses the severity of misophonia alone or misophonia comorbid with related auditory disorders.

The present study evaluated a Mandarin version of the S-Five, a self-report measure for symptoms of misophonia, within a Chinese sample. The S-Five was found to have comparable reliability and validity, and the five-factor structure found in the original English scale was replicated. The study provides further support that the S-Five is a reliable and valid tool for measuring symptoms of misophonia and that the Mandarin version can be used for the Chinese population.

## Data Availability Statement

The raw data supporting the conclusions of this article will be made available by the authors, without undue reservation.

## Ethics Statement

The study was conducted according to the guidelines of the Declaration of Helsinki, and approved by the Psychiatry, Nursing Midwifery Ethics Subcommittee of King’s College London (REC Reference Number: HR-19/20-17173). The patients/participants provided their written informed consent to participate in this study.

## Author Contributions

SV provided supervision to the project, completed the analysis, and contributed to the manuscript. JW carried out data collection and translation of the scales and contributed to the introduction and discussion sections of the manuscript. CH contributed to the data analysis and the manuscript. QW carried out data collection and translation of the scales. PS contributed to the data analysis. JG provided supervision to the project and contributed to the manuscript. All authors contributed to the article and approved the submitted version.

## Funding

SV and CH were funded or partially funded by the National Institute for Health and Care Research (NIHR) Maudsley Biomedical Research Centre at South London and Maudsley NHS Foundation Trust and King’s College London. This research was funded in whole, or in part, by the Wellcome Trust (JG; grant number 102176/B/13/Z).

## Conflict of Interest

The authors declare that the research was conducted in the absence of any commercial or financial relationships that could be construed as a potential conflict of interest.

## Publisher’s Note

All claims expressed in this article are solely those of the authors and do not necessarily represent those of their affiliated organizations, or those of the publisher, the editors and the reviewers. Any product that may be evaluated in this article, or claim that may be made by its manufacturer, is not guaranteed or endorsed by the publisher.
