# Supplementary material for: Evidence of Cross-Cultural Consistency of the S-Five Model for Misophonia: Psychometric Conclusions Emerging From the Mandarin Version
Source: Front Psychol. 2022 Jul 26;13:879881. doi: 10.3389/fpsyg.2022.879881 (PMC9361842; doi:10.3389/fpsyg.2022.879881)
Supplement: Supplementary file 1 [file Data_Sheet_1.docx]

# **Appendix**

**Appendix A1**. The S-Five in English, with scoring information

**Names are arranged by the alphabetic order of the first letter of first names*

| **A. The S-Five: Experience scale (S-Five -E)** |
| --- |
| Please read each statement carefully and base your answer on how true they feel to you based on your current thoughts, experiences, and reactions*: 0-not at all true to 10-completely true* |
| **Externalising** |
| People should not make certain sounds, even if they do not know about others' sensitivities |
| I get angry at other people because of how disrespectful they are with the noises they make |
| People should do everything they can to avoid making noises that might bother others |
| I react strongly to certain sounds because I cannot stand how selfish, thoughtless or bad-mannered people can be |
| Certain sounds are just bad manners, and it is not strange to feel intense anger about that |
| **Internalising** |
| The way I react to certain sounds makes me wonder whether deep inside I am just a bad person |
| The way I react to certain noises makes me feel like I must be an unlikable person deep down |
| I respect myself less because of my responses to certain sounds |
| I feel like I must be a very angry person inside because of the way I react to certain sounds |
| I dislike myself in the moments of my reactions to sounds |
| **Impact** |
| My job opportunities are limited because of my reaction to certain noises |
| I do not meet friends as often as I would like to because of the noises they make |
| There are places I would like to go but do not, because I am too worried about how the noises will impact me |
| I can see future where I cannot do everyday things because of my reactions to noises |
| The way I feel/react to certain sounds will eventually isolate me and prevent me from doing everyday things |
| **Outburst** |
| I can get so angry at certain noises that I get physically aggressive towards people to make them stop |
| Sometimes I get so distressed by noises that I use violence to try and make it stop |
| Some sounds are so unbearable that I will shout at people to make them stop |
| If people make certain sounds that I cannot bear, I become verbally aggressive |
| I am afraid I will do something aggressive or violent because I cannot stand the noise someone is making |
| **Threat** |
| I feel trapped if I cannot get away from certain noises |
| I feel anxious if I cannot avoid listening to certain sounds |
| If I cannot get away from certain noises, I am afraid I might panic or feel like I will explode |
| If I cannot avoid certain sounds, I feel helpless |
| I can experience distress as the result of some noises |
| *All items are rated in a 0-10 ordinal scale. Please randomise items before administering* |

**Scoring:**

In the S-Five-E, each item is rated in a 0-10 ordinal scale. Please add the responses of the corresponding items for each factor to compute the factor score /and all items for the total S-Five score. The factor scores range between 0 and 50, total score is between 0 and 250.

| B. The S-Five-T trigger checklist |
| --- |
| Trigger reaction items: Thinking about the past few weeks, what is the main feeling this sound* has caused you? *no feeling, irritation, distress, disgust, anger, panic, other feeling: negative, other feeling: positive, other: physiological reaction* |
|  |
| Trigger intensity items: Thinking about the past few weeks, please rate the intensity of your reaction to this sound* when made by another person or object *(from 0: doesn't bother me at all to 10: unbearable/causes suffering)* |
|  |
| *List of triggers currently included in the S-Five-t: Normal eating sounds, Certain letter sounds, Mushy foods being eaten, Sound of clipping nails, Swallowing, Keyboard tapping, Lip smacking, Normal breathing, Repetitive engine noises, Loud/unusual breathing, Mobile phone sounds, Repetitive coughing, Humming noise, Repetitive sniffing, Snoring, Certain accents, Whistling sound, Sound of tapping, Rustling, Chewing gum, Footsteps, Hiccups, Slurping, Cutlery noises, Sneezing, Certain words, Kissing, Joint cracking, Muffled sounds, Throat clearing, Baby crying, Repetitive barking, Loud chewing, Clock ticking, Crunching eating sounds, Teeth sucking, Yawning. |

The S-Five-t is made in a flexible format to allow researchers and treatment providers to customise the checklist according to the needs of their study/client. That is, its format facilitates adding or removing triggers as research findings progress or when treatment plans are being customised. More importantly, the format of the items allows to add or remove reactions.

Here we use 37 triggers and 9 reactions (no feeling to psychological reaction). We derive four useful summary indices from the S-Five-t checklist, according to the definitions and scoring guidelines described in Vitoratou et al. (2021b).

**Scoring:**

a) Trigger Count (TC) for each participant over all triggers: the index is computed by counting the number of non-zero responses in the trigger intensity items.

b) Reaction Count (RC) for each trigger over all participants: the index is computed for each reaction type separately, by counting over all participants the times a certain reaction was selected.

c) Frequency/Intensity of Reactions Score (FIRS): the index is computed by counting the trigger intensity items.

d) Relative Intensity of Reactions Score (RIRS): the index is computed by dividing the FIRS index by the trigger count TC, to derive an estimation of the intensity of the responses to triggers, relative to the number of triggers reported.

The scoring guide and the programming codes (SPSS, R project, Stata) to obtain all factors and indices are freely available upon request made to Silia Vitoratou ([silia.vitoratou@kcl.ac.uk](mailto:silia.vitoratou@kcl.ac.uk)).
