# Supplementary material for: Evidence of Cross-Cultural Consistency of the S-Five Model for Misophonia: Psychometric Conclusions Emerging From the Mandarin Version
Source: Front Psychol. 2022 Jul 26;13:879881. doi: 10.3389/fpsyg.2022.879881 (PMC9361842; doi:10.3389/fpsyg.2022.879881)
Supplement: Supplementary file 2 [file Data_Sheet_2.docx]

**Appendix A2**. The S-Five in Mandarin, with scoring information

*Translated by: Jingxin Wang, Qiaochu Wang*

| **A. S-5：经历量表（S-5-E）** |
| --- |
| ***请仔细阅读以下陈述，并根据您目前的想法，经历和反应，来判断这些陈述在多大程度上符合您的状况（0:一点也不符合；10:非常符合）*** |
| **外在反应** |
| 人们不应该弄出某些声音，即使他们不知道别人对这些声音的敏感程度 |
| 我会对那些弄出噪音的人生气，因为他们这种做法很不尊重他人 |
| 人们应该尽全力去避免制造出打扰他人的声音 |
| 对于一些声音我反应会很强烈, 因为我无法忍受制造这些声音的人有多么自私,不顾及他人以及没有礼貌 |
| 有的人弄出一些特定的声音是无理的行为，我对此感到强烈愤怒并不奇怪 |
| **内在反应** |
| 我对某些特定声音的反应让我怀疑我内心深处是不是个坏人 |
| 我对某些特定噪音的反应放我觉得我一定是一个不讨人喜欢的人 |
| 我的自尊会因为自己对某些声音的反应而减少 |
| 我对某些声音的反应让我觉得我本身一定是一个十分易怒的人 |
| 我在对某些声音反应的瞬间讨厌自己 |
| **日常影响** |
| 我对某些噪音反应限制了我的工作机会 |
| 我想经常和我的朋友们见面，但是我做不到，因为他们总是制造噪音 |
| 由于担心某些噪音对我的影响，我没办法去我去想去的地方 |
| 由于我对噪音的反应，我将无法做日常的事情 |
| 总有一天，我对某些声音的反应会让我孤立，使我无法做日常的事情 |
| **情绪爆发** |
| 我会对某些特定的噪音非常生气，以至于我会对制造这些声音的人进行身体攻击，让他们停下来 |
| 有的时候噪音会让我十分痛苦，所以我会尝试用暴力来使它停下 |
| 为了阻止有些让我忍无可忍的声音，我会向他人大吼 |
| 如果人们制造出一些让我无法忍受的声音，我会在语言上变得有攻击性 |
| 我害怕我会因为受不了其他人发出的噪音而做出有攻击性或者暴力的事情 |
| **受到威胁** |
| 如果我不能摆脱某些噪音，我会觉得我被困住了 |
| 如果我不能避免听到某些声音，我会觉得焦虑 |
| 如果我不能逃避某些声音，我怕我会感到恐慌或者要爆发 |
| 如果我不能避免某种声音，我会觉得无助 |
| 某些噪音会让我觉得痛苦 |
| ***所有陈述都按照0-10的顺序计分。请在使用前随机打乱陈述。*** |

计分：

每一个单项记分 0-10分

因素得分和总分：请将每个因素对应的单项的得分相加以计算每个因素的得分；将所有单项相加来计算S-5的总分。每个因素有五个对应单项，所以这些得分可以直接比较。

得分范围：每个因素的得分范围是0-50，总分范围是0-250。

| B.S-5: 触发音与反应量表（S-Five-t） |
| --- |
| 想一想在过去的几周里，这个声音主要带给您怎样的感觉? *没有感觉, 恼火, 痛苦, 恶心, 愤怒, 恐慌, 其他消极的感觉, 其他良好的感觉* |
|  |
| 请您评估一下在过去的几周里，当别人或者某些物体发出这种声音时，您的反应程度 （0:并不感到困扰；10:无法忍受/感到痛苦 |
|  |
| **S-5-T 目前包括的敏感声音：吃东西的声音，某些拼音字母的读音，吃糊状的食物的声音（例如布丁，慕斯或者是粥），剪指甲的声音，吞咽的声音，打字的声音，咂吧嘴的声音，平时呼吸的声音，重复的引擎噪音，大声的/不正常的呼吸声（鼻塞的声音，喘粗气），手机打字的声音或提示音，重复的咳嗽声，机器发出的嗡嗡声，重复用力吸鼻子的声音，打鼾的声音，某些特定的口音，吹口哨的声音，敲打东西的声音，塑料或纸的沙沙声，大声嚼口香糖的声音，脚步声，打嗝，吃东西或喝东西发出的呼哧声，使用餐具产生的噪音，打喷嚏的声音，某些字的读音，亲吻的声音，关节发出的响声，透过墙壁，天花板，耳机传来的含糊的声音，清嗓子的声音，婴儿啼哭的声音，重复的犬吠声，大声咀嚼食物的声音，表走针的声音，吃松脆食物的声音 (吃苹果，胡萝卜，薯片或其他脆的食物的声音)，吸牙缝的声音，打哈欠的声音* |

S-Five-t (敏感声音与反应量表)

S-Five-t采用了灵活的格式，允许研究人员和治疗机构根据他们的研究/客户的需要定制检查表。也就是说，它的格式便于在研究结果进展时或在定制治疗计划时增加或删除触发因素。更重要的是，项目的格式允许添加或删除反应。

在这里，我们使用37个诱因（敏感声音）和9个反应（对心理反应没有感觉）。根据Vitoratou等人（2021b）中描述的定义和评分准则，我们从S-Five-t检查表中得出四个有用的总结指数

a) 敏感声音数量（TC）：该指数是通过计算触发敏感声音中的非零响应数量来计算的。

b) 每一个情绪的情绪反应得分（RC）：通过计算所有参与者选择某种反应的次数，对每种反应类型分别计算指数。

c)反应频率/强度得分（FIRS指数）：该指数是通过计算对敏感声音的反应强度来计算的。

d)敏感声音相对强度 （RIRS指数）：该指数的计算方法是用FIRS指数除以TC，以得出相对于报告的敏感声音数量而言，对敏感声音反应强度的估计

评分指南和获得所有因素和指数的编程代码（SPSS、R项目、Stata）可向Silia Vitoratou免费索取 ([silia.vitoratou@kcl.ac.uk](mailto:silia.vitoratou@kcl.ac.uk))。
