# Supplementary material for: Evidence of Cross-Cultural Consistency of the S-Five Model for Misophonia: Psychometric Conclusions Emerging From the Mandarin Version
Source: Front Psychol. 2022 Jul 26;13:879881. doi: 10.3389/fpsyg.2022.879881 (PMC9361842; doi:10.3389/fpsyg.2022.879881)
Supplement: Supplementary file 3 [file Data_Sheet_3.docx]

# **Appendix**

**Appendix A3.** Exploratory factor analysis pattern matrix for the S-Five.

| **Table A3**  *Exploratory factor analysis pattern matrix of the 5 -factor solution of the 25 S-Five items (N=225)* | | | | | |
| --- | --- | --- | --- | --- | --- |
| **S-Five**  **Items per factor** | **Externalising** | **Internalising** | **Impact** | **Threat** | **Outburst** |
| ***Externalising*** | | | | | |
| I06 Others avoid noises | 0.709 | -0.061 | -0.041 | 0.100 | -0.069 |
| I13 Others not make sounds | 0.687 | 0.001 | 0.079 | -0.037 | 0.083 |
| I16 Others selfish | 0.807 | 0.088 | 0.101 | -0.034 | 0.002 |
| I21 Others bad manners | 0.791 | -0.011 | 0.052 | -0.059 | 0.056 |
| I25 Others disrespectful | 0.794 | -0.027 | -0.036 | 0.132 | -0.053 |
| **Internalising** | | | | | |
| I05 Respect myself less | -0.088 | 0.784 | 0.209 | -0.005 | -0.032 |
| I08 Unlikeable person | 0.052 | 0.781 | -0.042 | -0.015 | 0.169 |
| I12 Angry person inside | 0.072 | 0.579 | -0.155 | 0.125 | 0.307 |
| I18 Bad person inside | 0.070 | 0.800 | 0.017 | -0.001 | -0.012 |
| I19 Dislike self | -0.088 | 0.784 | 0.209 | -0.005 | -0.032 |
| **Impact** | | | | | |
| I01 Do not meet friends | 0.073 | 0.111 | 0.781 | -0.023 | 0.036 |
| I09 Eventually isolated | 0.088 | 0.159 | 0.634 | 0.086 | 0.051 |
| I14 Avoid places | 0.054 | 0.068 | 0.747 | 0.091 | 0.044 |
| I15 Cannot do things | 0.093 | -0.006 | 0.805 | -0.019 | 0.038 |
| I20 Limited job opportunities | -0.082 | -0.036 | 0.796 | 0.128 | 0.060 |
| **Outburst** | | | | | |
| I04 Verbally aggressive | 0.268 | 0.044 | -0.131 | 0.174 | 0.590 |
| I17 Physically aggressive | -0.093 | 0.051 | 0.267 | 0.020 | 0.620 |
| I22 Violence | -0.017 | 0.106 | 0.195 | 0.066 | 0.606 |
| I23 Shout at people | 0.013 | -0.009 | 0.032 | 0.112 | 0.705 |
| I24 Afraid of outburst | 0.034 | 0.201 | 0.169 | 0.007 | 0.618 |
| ***Threat*** | | | | | |
| I02 Panic or explode | -0.044 | -0.02 | 0.105 | 0.811 | 0.092 |
| I03 Feel helpless | -0.001 | -0.004 | 0.104 | 0.766 | 0.049 |
| I07 Feel anxious | 0.03 | 0.025 | 0.003 | 0.89 | -0.095 |
| I10 Experience distress | 0.132 | 0.024 | -0.067 | 0.737 | 0.067 |
| I11 Feel trapped | -0.013 | 0.061 | -0.016 | 0.827 | 0.022 |
| *Oblimin rotation* | | | | | |
